# The Medical Profession in Paris and London

**Published:** 1847-12

**Authors:** 


*The Medical Profession in Paris and London.*—According to M. Domange's Medical Almanac, the number of practitioners in Paris 1st. Jan. 1847; amounted to

| | | |
|---|---|---|
| Doctors of Medicince - - | 1442 | |
| Officers de Sante - - - - | 165 | |
| | | 1617 |
| To these may be added, Pharmaciens - - - - - | 345 | |
| Midwives - - - - - - - | 480 | |
| Total - - - - - - | | 2442 |

In estimating the amount of practice which falls to the lot of these 1617 qualified medical men (at Paris as elsewhere, charlatans and unlicensed persons abound likewise,) we find tho official statements in 1846 give us a population (including, however, hospitals colleges, and garrison,) of 1,053,097 souls. In 1845, of 32,705 birth, 5,678 occurred in the hospitals, that is, more than a sixth part did not fall into the hands of the practitioners and midwives, these last taking the lion's share of the remainder. In the same year 25,156 deaths took place, of which 15,888 took place at their own houses, the rest dying in hospitals, &c. In 1845 there were 1598 practitioners, and dividing the deaths among these, we find about 10 must fall to the lot of each. In the hospitals, the proportion of deaths to patients is about one-eighth; but if, in the easier classes of society, we call it a fifteenth, each practitioner would then, upon an average, have an annual practice among 150 patients, or 12 per month. We may leave to others to calculate the mean number of visits these may require, and the product of such, at charges of 5, 3, 2 francs, or even less—to say nothing of bad debts. This, if patients were equally divided.; but there are some few of great repute who receive their £4000, others whom the hardness of the times leaves only £2000; and so on, from step to step, to those, who, yet better provided for than the mass of their brethren, with great difficulty attain £200 at the end of the year. Lower, and much lower still, there are practitioners who make as litsle as £60, £50, or £40 per annum; and how many are there who in the first year of their practice do not take £20.

And yet the evil is on the increase: the number of practitioners is constantly augmenting. In 1845 there were but 1430 doctors: 50 died and 62 left; but this vacuum of 112 was not only filled up, but replaced by 124. In 1845 there were 168 *officiers de sante*, 175 in 1847: so that the total addition of practitioners in only two years amounted to 19. In 1845 there were 450 midwives, in 1847 there are 480; while the accouchments at home only amounted to 27,227. Take from these the gratuitous confinements provided by the charitable societies, and those conducted by the practitioner, and there certainly cannot remain a mean of 50 patients per annum for each midwife. The majority then cannot live by their profession. What then do they live by? That would be worth while inquiring into?—*L'Union Medicale*, No. 102.

[The writer goes on to recommend the limitation of the number of the practitioners to the wants of the population; forgetting apparently that every occupation and profession is in like manner overburdened and could put forth equal claims to repression, if these were not perfectly inadmissible in the present state of society, which is to be governed by moral and prudential restriction, and not by the exclusivism of a worn-out period. The medical profession has, however, the remedy in its own hands in a great measure by means of which it may right itself, and yet benefit the public, namely, the increasing the period of study and the amount of requisite qualifications. Certainly the pecuniary position of the medical body in Paris, if the above representation be correct, is far worse than that of our own capital; since, for our own two million souls, we have but 2,413 qualified practitioners, while for their own million they have 1617. Then again, the midwives with us are a most insignificant class, employed only by the poorest of the population as a matter of necessity; while too many of our own practitioners add to their strictly professional receipts, the profit of tradesmen by vending drugs, perfumery, ann even quack medicines! For our parts we prefer the honorable poverty of our neighbors to this lurative but degrading practice among ourselves].—*Medical Chir. Review.*

